# Measles and Secondary Hemophagocytic Lymphohistiocytosis

**DOI:** 10.3201/eid1809.120235

**Published:** 2012-09

**Authors:** Chiara Iaria, Maria Silvana Leonardi, Agata Buda, Maria Luisa Toro, Antonio Cascio

**Affiliations:** Azienda Ospedaliera Piemonte-Papardo, Messina, Italy (C. Iaria);; and Policlinico “G. Martino” University Hospital, Messina (M.S. Leonardi, A. Buda, M.L. Toro, A. Cascio)

**Keywords:** Measles, pneumonia, hemophagocytosis, pancytopenia, secondary hemophagocytic lymphohistiocytosis, viruses

**To the Editor:** We found interesting the article by Lupo et al. about a case of fatal measles in an immunocompetent 29-year-old woman (Fatal measles without rash in immunocompetent adult, France; http://dx.doi.org/10.3201/eid1803.111300). Perhaps, however, the possible diagnosis of secondary hemophagocytic lymphohistiocytosis (HLH) should also have been considered in that setting.

HLH is a potentially fatal hyperinflammatory syndrome characterized by histiocyte proliferation and hemophagocytosis. HLH may be inherited (i.e., primary, familial, generally occurring in infants) or may occur at any age secondary to infection, malignancy, or rheumatologic disease. Secondary HLH is determined according to clinical criteria from the HLH Study Group of the Histiocyte Society, which require >5 of the following for a diagnosis: fever; splenomegaly; cytopenia (affecting >2 cell lineages); hypertriglyceridemia or hypofibrinogenemia; hemophagocytosis in the bone marrow, spleen, or lymph nodes; low or absent natural killer cell cytotoxicity; hyperferritinemia; and elevated levels of soluble CD25.

We conducted a PubMed search and found 5 articles that described 6 cases of HLH in patients with measles (*1*–*5*). Pneumonia was described in all of them (*1*–*5*), and central nervous system involvement was described in 3 (*1*,*4*). Four cases occurred in children, 3 of them immunocompetent (*1*,*3*–*5*). The 2 adults were an immunocompetent 18-year-old man who had acute respiratory distress (*2*) and a 19-year-old man with acute lymphocytic leukemia who had measles pneumonia and acute hemorrhagic leukoencephalitis (*1*). The only fatal case occurred in an immunocompromised 8-year-old boy with giant-cell pneumonia (*3*).

The identification of hemophagocytosis in bone marrow aspirate represents only 1 of the 5–8 criteria needed for a diagnosis of HLH; conversely, a bone marrow aspirate lacking hemophagocytosis does not rule out the diagnosis of HLH. Still, we believe HLH should be considered for any patient with fever and pancytopenia, especially in the presence of respiratory distress or multiorgan dysfunction. An appropriate therapy could save the patient (Secondary hemophagocytic syndrome in adults: a case series of 18 patients in a single institution and a review of literature; http://dx.doi.org/10.1002/hon.960).
