# Supplementary material for: Measles and Secondary Hemophagocytic Lymphohistiocytosis
Source: Emerg Infect Dis. 2012 Sep;18(9):1529. doi: 10.3201/eid1809.120235 (PMC3437716; doi:10.3201/eid1809.120235)
Supplement: Related texts

# Reply

**DOI:** 10.3201/eid1809.120749

**Authors:** Julien Lupo, Jean-François Timsit, Patrice Morand

**Affiliations:** Centre Hospitalier Universitaire de Grenoble and Université Joseph Fourier, Grenoble, France

**In Response:** We thank Iaria et al. (*1*) for their comments on our letter reporting an unusual case of fatal measles without rash in an immunocompetent woman who manifested cytopenias and an intractable acute respiratory distress syndrome (*2*). The authors suggest that secondary hemophagocytic lymphohistiocytosis (HLH) could have been considered in this patient. Our reply supplies supplementary clinical and laboratory findings that could be useful for discussion.

During our patient’s hospitalization, we were able to investigate 6/8 diagnostic criteria for HLH proposed by the Histiocyte Society (*3*). Of these, only 2 or 3 were found: persistent fever at 38.5°C; hypertriglyceridemia at 267 mg/dL (analysis performed at day 7); and cytopenias, which preferentially affected erythrocytes and lymphocytes. (Thrombocytopenia was moderate at >100 × 10^9^ platelets/L, and no neutropenia was found [>2 × 10^9^ neutrophils/L].) Liver function was not affected; no hepatomegaly was found, and alanine aminotransferase, aspartate aminotransferase, and bilirubin levels remained within reference ranges. Physical examinations did not detect splenomegaly, and laboratory findings did not show hypofibrinogenemia or ferritin level exceeding 500 ng/mL. A bone marrow biopsy performed on day 2 of hospitalization did not show hemophagocytosis. Studies of natural killer cell function and soluble CD25, which are also proposed diagnostic criteria for HLH, were not performed.

Overall, we found that the arguments in favor of HLH were too limited to consider this diagnosis and initiate an aggressive therapeutic approach based on immunosuppressive drugs. Even if, in the event of HLH, an early and appropriate treatment can be life-saving, the destruction of the remaining immune functions might also be lethal for the patient.

## References

[1] Iaria C, Leonardi MS, Buda A, Toro ML, Cascio A. Measles and secondary hemophagocytic lymphohistiocytosis [letter]. Emerg Infect Dis. 2012;18:1529. 10.3201/eid1809.12023522931858PMC3437716

[2] Lupo J, Bernard S, Wintenberger C, Baccard M, Vabret A, Antona D, Fatal measles without rash in Immunocompetent adult, France. Emerg Infect Dis. 2012;18:521–3. 10.3201/eid1803.11130022377448PMC3309583

[3] Janka GE. Familial and acquired hemophagocytic lymphohistiocytosis. Annu Rev Med. 2012;63:233–46. 10.1146/annurev-med-041610-13420822248322
